# Cardiometabolic risk factors as determinants of peripheral nerve function: the Maastricht Study

**DOI:** 10.1007/s00125-020-05194-5

**Published:** 2020-06-15

**Authors:** Jeroen H. P. M. van der Velde, Annemarie Koster, Elsa S. Strotmeyer, Werner H. Mess, Danny Hilkman, Jos P. H. Reulen, Coen D. A. Stehouwer, Ronald M. A. Henry, Miranda T. Schram, Carla J. H. van der Kallen, Casper G. Schalkwijk, Hans H. C. M. Savelberg, Nicolaas C. Schaper

**Affiliations:** 1 Department of Nutrition and Movement Sciences, Maastricht University, P.O. Box 616, 6200 MD Maastricht, the Netherlands; 2 NUTRIM School of Nutrition and Translational Research in Metabolism, Maastricht University, Maastricht, the Netherlands; 3 Department of Internal Medicine, Maastricht University Medical Center+, Maastricht, the Netherlands; 4 CARIM School for Cardiovascular Diseases, Maastricht University, Maastricht, the Netherlands; 5 Department of Social Medicine, Maastricht University, Maastricht, the Netherlands; 6 CAPHRI Care and Public Health Research Institute, Maastricht University, Maastricht, the Netherlands; 7 Department of Epidemiology, Graduate School of Public Health, University of Pittsburgh, Pittsburgh, PA USA; 8 Department of Clinical Neurophysiology, Maastricht University Medical Center+, Maastricht, the Netherlands; 9 Heart and Vascular Centre, Maastricht University Medical Centre+, Maastricht, the Netherlands

**Keywords:** Cardiometabolic risk factors, Diabetes status, Electrophysiological, Nerve conduction test, Neuropathy, The metabolic syndrome

## Abstract

**Aims/hypothesis:**

We aimed to examine associations of cardiometabolic risk factors, and (pre)diabetes, with (sensorimotor) peripheral nerve function.

**Methods:**

In 2401 adults (aged 40–75 years) we previously determined fasting glucose, HbA_1c_, triacylglycerol, HDL- and LDL-cholesterol, inflammation, waist circumference, blood pressure, smoking, glucose metabolism status (by OGTT) and medication use. Using nerve conduction tests, we measured compound muscle action potential, sensory nerve action potential amplitudes and nerve conduction velocities (NCVs) of the peroneal, tibial and sural nerves. In addition, we measured vibration perception threshold (VPT) of the hallux and assessed neuropathic pain using the DN4 interview. We assessed cross-sectional associations of risk factors with nerve function (using linear regression) and neuropathic pain (using logistic regression). Associations were adjusted for potential confounders and for each other risk factor. Associations from linear regression were presented as standardised regression coefficients (*β*) and 95% CIs in order to compare the magnitudes of observed associations between all risk factors and outcomes.

**Results:**

Hyperglycaemia (fasting glucose or HbA_1c_) was associated with worse sensorimotor nerve function for all six outcome measures, with associations of strongest magnitude for motor peroneal and tibial NCV, *β*_fasting glucose_ = −0.17 SD (−0.21, −0.13) and *β*_fasting glucose_ = −0.18 SD (−0.23, −0.14), respectively. Hyperglycaemia was also associated with higher VPT and neuropathic pain. Larger waist circumference was associated with worse sural nerve function and higher VPT. Triacylglycerol, HDL- and LDL-cholesterol, and blood pressure were not associated with worse nerve function; however, antihypertensive medication usage (suggestive of history of exposure to hypertension) was associated with worse peroneal compound muscle action potential amplitude and NCV. Smoking was associated with worse nerve function, higher VPT and higher risk for neuropathic pain. Inflammation was associated with worse nerve function and higher VPT, but only in those with type 2 diabetes. Type 2 diabetes and, to a lesser extent, prediabetes (impaired fasting glucose and/or impaired glucose tolerance) were associated with worse nerve function, higher VPT and neuropathic pain (*p* for trend <0.01 for all outcomes).

**Conclusions/interpretation:**

Hyperglycaemia (including the non-diabetic range) was most consistently associated with early-stage nerve damage. Nonetheless, larger waist circumference, inflammation, history of hypertension and smoking may also independently contribute to worse nerve function.

**Electronic supplementary material:**

The online version of this article (10.1007/s00125-020-05194-5) contains peer-reviewed but unedited supplementary material, which is available to authorised users.

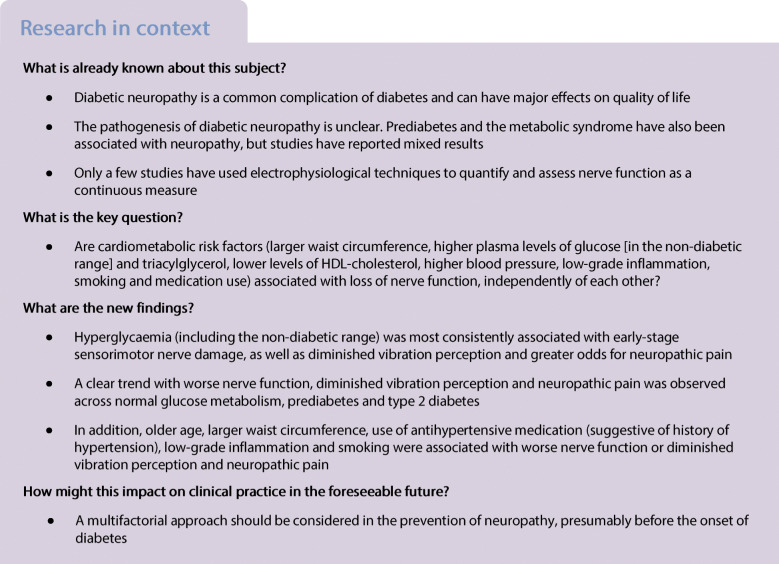



## Introduction

Diabetic neuropathy is one of the most common complications of diabetes mellitus [1], and a major cause of reduced quality of life, gait disturbances, foot ulceration, fall-related injuries and disability [2]. During their lifetimes, up to 50% of patients with type 2 diabetes are affected by some form of neuropathy, of which distal symmetric polyneuropathy is most common [3, 4]. Moreover, neuropathy was already present in 10–20% of patients at the time of diagnosis of type 2 diabetes [5], suggesting that neuropathy is initiated in early stages of the pathogenesis of diabetes. Indeed, studies have demonstrated that neuropathy is present in the prediabetic stage [6–8], although not consistently [9, 10].

Traditionally, it has been suggested that hyperglycaemia is the main driver of microvascular damage and subsequent neuropathy. Therefore, glycaemic control is considered fundamental in its prevention [11]. However, a study in patients with type 2 diabetes showed that the aggregation of components for the metabolic syndrome was significantly associated with sensory neuropathy [12]. In subsequent studies, the metabolic syndrome has been associated with neuropathy regardless of the presence of (pre)diabetes [13–17], but not consistently [7].

As increased blood glucose levels, even in the non-diabetic range, as well as other cardiometabolic risk factors could contribute to microvascular dysfunction, we postulated that each of these factors contributes to a progressive decline of nerve function, before the development of type 2 diabetes or overt neuropathy. To examine this, studies are needed that do not dichotomise risk factors (such as the presence of the metabolic syndrome) or dichotomise outcomes (such as the presence of neuropathy), but that analyse risk factors and outcomes as continuous variables. In addition, estimates of the prevalence of neuropathy may vary depending on the methods used, which may have contributed to the discrepancies in reported associations between metabolic risk markers and neuropathy [7, 18, 19]. Assessing nerve function as a continuous measure with objective electrophysiological techniques may therefore be more relevant to study the aforementioned associations [18]. However, such population-based studies are scarce and mainly focus on components of the metabolic syndrome [14, 20].

In light of the above, our aim was to examine the associations of multiple classical and newer cardiometabolic risk factors and mildly elevated blood glucose levels (such as in prediabetes [i.e. impaired fasting glucose and/or impaired glucose tolerance]) with measures of motor and sensory nerve function assessed by electrophysiological techniques in a large, population-based cohort: the Maastricht Study. In addition, we assess their associations with clinical measures such as vibration perception threshold (VPT) and neuropathic pain. We hypothesised that unfavourable cardiometabolic risk and elevated blood glucose levels within the prediabetic range are associated with impaired nerve function, independently of fasting glucose and of each other.

## Methods

### Population

We used data from the Maastricht Study, an observational, prospective, population-based cohort study. The rationale and methodology have been described previously [21] (also see electronic supplementary material [ESM] Methods). The present report includes cross-sectional data from 3451 participants, who completed the baseline survey between November 2010 and September 2013. The study complies with the Declaration of Helsinki and has been approved by the institutional medical ethics committee (NL31329.068.10) and the Minister of Health, Welfare and Sports of the Netherlands (Permit 131088-105234-PG). All participants gave written, informed consent.

### Risk factors

We considered the following cardiometabolic risk factors: age, fasting glucose, HbA_1c_, 2 h post-load glucose (for additional analyses), triacylglycerol, HDL- and LDL-cholesterol, waist circumference, inflammation, office systolic blood pressure (24 h blood pressure for additional analyses) and diabetes status. In addition, we considered smoking, lipid-modifying and antihypertensive medications, and the metabolic syndrome. Details of assessments have been previously described [21]. Inflammation markers were measured in plasma and included high-sensitivity C-reactive protein (CRP), serum amyloid A (SAA), IL-6, IL-8, TNF-α and soluble intercellular adhesion molecule-1 (sICAM-1). These were converted into a sum-score for analyses, calculated by summation of the individual *z* scores of inflammation markers. Such a summary score predicted future cardiovascular events and mortality in earlier studies [22]. Use of medication was assessed during a medication interview. Smoking behaviour was derived from a questionnaire.

To determine diabetes status, all participants (except those who used insulin) underwent an OGTT after an overnight fast [21]. Participants were categorised according to the World Health Organization 2006 criteria [23] into normal glucose metabolism (NGM), prediabetes (impaired fasting glucose and/or impaired glucose tolerance) or type 2 diabetes. The metabolic syndrome was defined according to the Adult Treatment Panel (ATPIII) guidelines [24] (see ESM Methods).

### Nerve conduction study

Nerve function of the lower limbs was assessed with a Medelec Synergy electromyography apparatus (version 15.0, Viasys Healthcare, UK) using surface electrodes. Before testing, feet and lower legs were warmed in warm water (38°C) for a minimum duration of 10 min, to ensure that skin temperature (measured on the dorsal surface of the foot) was >32°C. Motor peroneal and tibial nerves and sensory sural nerve were examined at supra-maximal stimulation.

Peroneal nerve function was recorded on the right leg, at the digitorum brevis muscle with stimulations at the ankle (8 cm proximal from the recording site), below the fibular head and above the fibular head. Tibial nerve function was recorded on the left leg, at the abductor hallucis muscle with stimulations at the ankle and in the popliteal fossa. Sural nerve function was recorded on the left leg between the lateral malleolus and the Achilles tendon while stimulating 12 cm proximal to the recording site.

Variables analysed were compound muscle action potential (CMAP) amplitudes (stimulated at the ankle), nerve conduction velocities (NCV) of the peroneal and tibial nerves, and the sural sensory nerve action potential (SNAP) amplitude and NCV.

### Peripheral vibration perception

Peripheral VPT was tested by use of a Horwell Neurothesiometer (Scientific Laboratory Supplies, Nottingham, UK). Vibration thresholds were tested three times at the distal phalanx of the hallux on both feet. Mean threshold was calculated for each foot and the highest mean threshold was used for analyses.

### Neuropathic pain

Neuropathic pain was defined as a score ≥3 on the DN4 interview [25].

### Covariates

Questionnaires were used to collect information on age, sex, educational level, alcohol consumption (high consumer [women >7 glasses per week; men >14 glasses per week]), cardiovascular disease history (see ESM Methods) and mobility limitations (defined as having difficulty walking 500 m or climbing stairs). Kidney function (estimated glomerular filtration rate [in ml min^−1^ 1.73 m^−2^]) was calculated from serum creatinine and cystatin [26].

### Statistical analyses

First, population characteristics and measures of nerve function were described for the total population and by tertiles of sural SNAP amplitude using the appropriate descriptive statistics.

Second, associations between cardiometabolic risk factors and nerve function were examined with standardised linear regression analyses. All continuous risk factors and the six outcomes of nerve function were standardised to *z* scores (with a mean of 0 and an SD of 1) in order to compare the magnitudes of observed associations between all risk factors and outcomes (see ESM methods for details). Two models were fitted with covariates that we selected a priori. In the first model, associations were adjusted for age, sex, height, educational level, skin temperature at start of nerve function assessment and heating time. In the second model, all associations were additionally adjusted for all other risk factors as well as alcohol intake, cardiovascular disease history, mobility limitations and kidney function. In addition, a composite score for nerve function was calculated as the mean of *z* scores of individual measures of nerve function. A composite score is considered to be more sensitive and reproducible for detection of peripheral neuropathy than individual attributes of nerve conduction [27], and we report this score to summarise the associations with nerve conduction outcomes. Associations were expressed as standardised regression coefficients (*β*) with 95% CIs.

For undetectable sural nerve responses (*n* = 165), the likelihood of an absent response (OR with 95% CI) was calculated using logistic regression analyses using similar adjustments as described above.

Third, we examined the associations of prediabetes and type 2 diabetes with nerve function. Associations were adjusted for age, sex, height, waist circumference, inflammation, smoking, alcohol intake, cardiovascular disease history, mobility limitations, skin temperature at start of nerve function assessment and heating time. To test for a linear trend across NGM, prediabetes and type 2 diabetes, glucose metabolism status was categorised (NGM = 0, prediabetes = 1 and type 2 diabetes = 2) and used in the linear regression models.

Fourth, we examined the associations of cardiometabolic risk factors with VPT (linear regression) and with neuropathic pain (logistic regression) in similar models as described above.

In addition, the associations of the metabolic syndrome (overall) and the number of criteria for the metabolic syndrome (3, 4 or 5 criteria vs 0–2 criteria) with nerve function were examined.

Potential interaction effects of sex and of type 2 diabetes were assessed by computing interaction terms (sex × risk factor and type 2 diabetes × risk factor) and adding these (separately) in the fully adjusted models. No interaction effect of sex was observed. Overall, analyses stratified on type 2 diabetes yielded non-significant differences, except for inflammation (see below). Therefore, we present the analyses for the total population in the main manuscript and stratified analyses are presented in the ESM.

All analyses were performed using SPSS version 25.0 (IBM Corp, Armonk, NY, USA).

## Results

### Population

Data were available for 2401 participants. A flow diagram with details is provided in ESM Fig. 1. Compared with participants included in this study, those excluded had a similar distribution of sex, but were older, had a higher BMI and more often had type 2 diabetes (ESM Table 1).

In Table 1, the population characteristics are provided for the total population and by tertiles of sural SNAP amplitude. Compared with those in the highest tertile, those in the lowest tertile were older, more often male, had elevated levels of multiple cardiovascular risk factors, had higher prevalence of the metabolic syndrome and type 2 diabetes, and reported more frequently the use of medication.

**Table 1:** Population characteristics of the total population and by tertiles of sural SNAP amplitude

Characteristic	Total (*n* = 2401)	High (*n* = 793)	Medium (*n* = 796)	Low (*n* = 812)
Age (years)	59.3 (8.2)	56.4 (8.2)	59.4 (7.9)	62.0 (7.5)
Sex (% men)	51.1	41.5	52.6	58.9
Education level (% high)	39.7	39.2	42.6	37.3
Smoking status (% current smokers)	12.8	12.4	14.1	11.8
Alcohol use (% high consumers)	26.7	26.7	25.8	27.6
Mobility limitation (% with limitation)	2.5	2.1	2.1	3.3
(History of) cardiovascular disease (%)	16.1	12.9	15.7	19.3
The metabolic syndrome (%)	37.1	26.9	38.8	45.3
Prediabetes (%)	15.4	13.2	16.7	16.3
Type 2 diabetes (%)	25.3	16.8	24.4	34.5
Fasting glucose (mmol/l)	5.5 [5.0–6.3]	5.3 [4.9–5.8]	5.5 [5.1–6.3]	5.8 [5.3–7.3]
HbA_1c_ (mmol/mol)	38.0 [35.0–43.0]	37.0 [34.0–40.0]	38.0 [35.0–43.0]	39.5 [36.0–47.0]
HbA_1c_ (%)	5.6 [5.3–6.0]	5.5 [5.3–5.8]	5.6 [5.3–6.1]	5.7 [5.4–6.5]
BMI (kg/m^2^)	26.8 (4.3)	26.0 (3.8)	26.8 (4.2)	27.5 (4.6)
Waist circumference (cm)	94.9 (13.1)	91.6 (12.1)	95.1 (12.4)	98.0 (13.7)
Systolic blood pressure (mmHg)	134.6 (17.9)	131.7 (17.8)	134.9 (17.2)	137.2 (18.2)
Diastolic blood pressure (mmHg)	76.2 (9.8)	75.9 (10.2)	76.7 (9.6)	76.0 (9.7)
HDL-cholesterol (mmol/l)	1.5 (0.5)	1.6 (0.5)	1.5 (0.5)	1.5 (0.5)
LDL-cholesterol (mmol/l)	3.1 (1.0)	3.1 (1.0)	3.2 (1.0)	3.0 (1.0)
Triacylglycerol (mmol/l)	1.2 [0.9–1.7]	1.2 [0.9–1.6]	1.2 [0.9–1.7]	1.2 [0.9–1.7]
C-reactive protein (μg/ml)	1.2 [0.6–2.6]	1.1 [0.6–2.5]	1.2 [0.6–2.6]	1.2 [0.6–2.7]
Serum amyloid A (μg/ml)	3.1 [2.0–5.3]	3.2 [2.0–5.4]	3.0 [1.9–5.1]	3.1 [2.1–5.3]
sICAM-1 (ng/ml)	335.6 [289.5–394.7]	330.3 [280.3–392.1]	333.4 [292.0–388.6]	340.7 [293.5–401.8]
IL-6 (pg/ml)	0.6 [0.4–0.9]	0.5 [0.3–0.8]	0.6 [0.4–0.9]	0.6 [0.4–1.0]
IL-8 (pg/ml)	4.1 [3.3–5.2]	3.8 [3.1–4.8]	4.1 [3.3–5.2]	4.4 [3.5–5.6]
TNF-α (pg/ml)	2.2 [1.9–2.5]	2.1 [1.8–2.4]	2.1 [1.9–2.5]	2.5 [2.0–2.7]
Glucose-lowering medication (%)	19.0	12.4	18.5	26.0
Antihypertensive medication (%)	37.6	29.6	34.7	48.2
Lipid-lowering medication (%)	34.2	25.9	33.3	43.3
VPT (V)	11.5 [8.0–17.7]	9.3 [6.7–13.2]	11.8 [8.3–17.0]	14.7 [9.5–22.3]
Neuropathic pain (%)	5.7	4.4	5.4	7.3
Sural SNAP amplitude, range (μV)^a^	undetectable – 41.6	11.6–41.6	6.7–11.6	undetectable – 6.6
Sural NCV (m/s)^a^	48.4 (5.8)	49.5 (5.2)	48.2 (5.7)	47.0 (6.1)
Peroneal CMAP amplitude (mV)	5.0 (2.1)	5.5 (2.1)	5.1 (2.1)	4.5 (2.0)
Peroneal NCV (m/s)	45.9 (4.7)	47.2 (.1)	46.0 (4.4)	44.1 (5.0)
Tibial CMAP amplitude (mV)	9.7 (4.4)	11.2 (4.3)	9.8 (4.1)	8.2 (4.2)
Tibial NCV (m/s)	44.0 (4.8)	45.3 (4.6)	44.1 (4.3)	42.6 (5.0)

Values are expressed as mean (SD), median [25th–75th percentile] or percentages, unless indicated otherwise

Tertiles of sural SNAP amplitudes were derived from data from the current study and do not represent clinical cut-off values

^a^*n* = 2236 due to omission of undetectable sural response

### Age

**Fig. 1:**
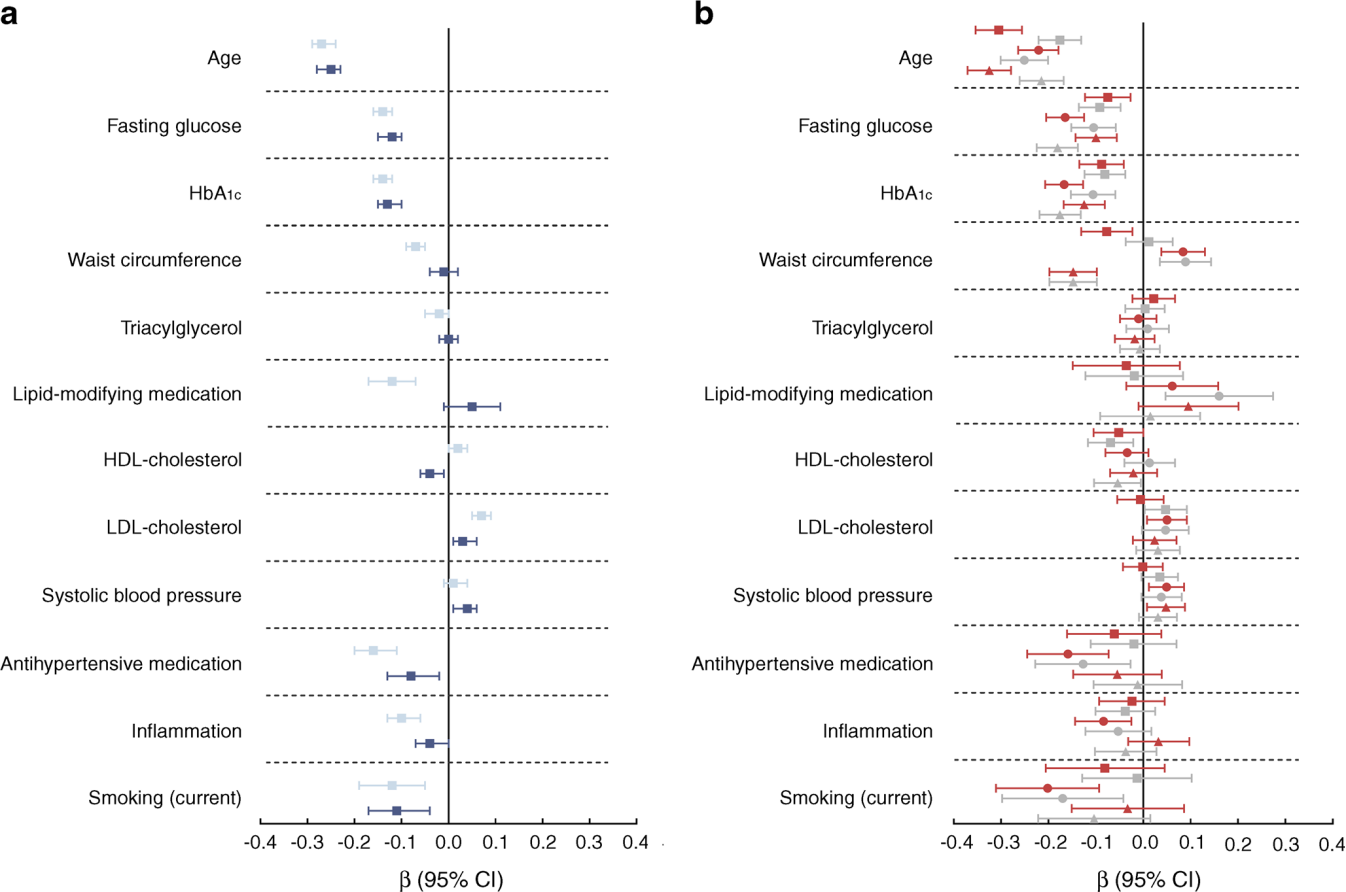
Standardised associations (expressed as *β* with 95% CIs) of cardiometabolic risk factors and nerve function. (**a**) Associations with the sum-score of nerve function. Associations in model 1 (light blue) were adjusted for sex, height, age (with the exception of associations of age), educational level and skin temperature. Associations in model 2 (dark blue) were additionally adjusted for alcohol consumption, mobility limitations, CVD (history) and kidney function. In addition, all associations in model 2 were adjusted for each of the other risk factors with multivariate regression, with the exception of HbA_1c_. Further, HbA_1c_ was not adjusted for fasting glucose. (**b**) Associations with individual measures of nerve function. Red squares represent sural SNAP amplitude, grey squares represent sural NCV, red circles represent peroneal CMAP amplitude, grey circles represent peroneal NCV, red triangles represent tibial CMAP amplitude and grey triangles represent tibial NCV. Associations are adjusted as in model 2 in (**a**)

**Fig. 2:**
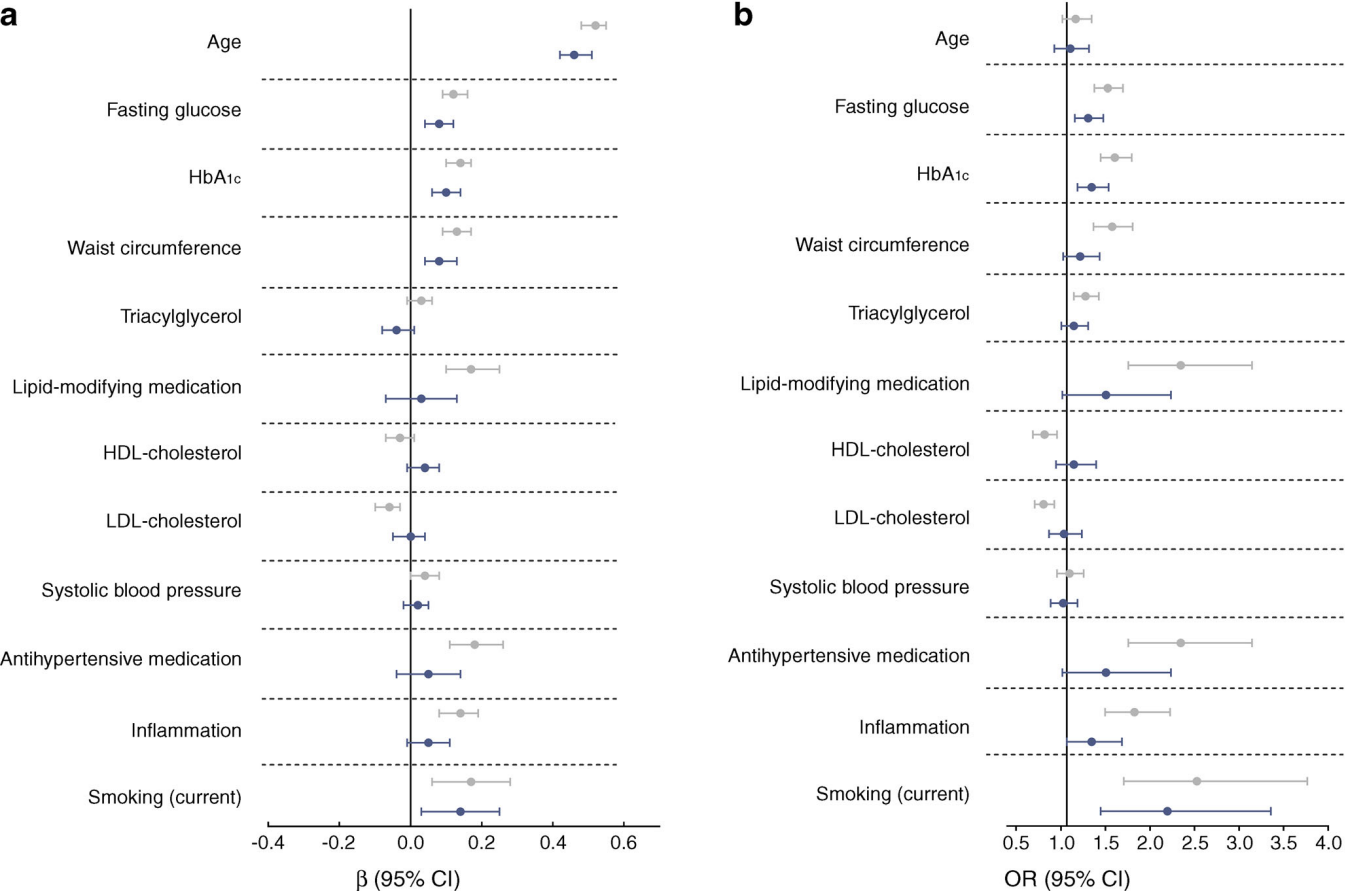
(**a**) Standardised associations (expressed as *β* with 95% CIs) of cardiometabolic risk factors and VPT. A higher threshold indicates worse score. (**b**) Standardised associations (expressed as ORs with 95% CIs) of cardiometabolic risk factors and neuropathic pain (OR >1 indicates greater likelihood for neuropathic pain). Associations in model 1 (grey) were adjusted for age, sex, height, educational level and skin temperature. Associations in model 2 (dark blue) were additionally adjusted for alcohol consumption, mobility limitations, CVD (history) and kidney function. In addition, all associations in model 2 were adjusted for each of the other risk factors with multivariate regression, with the exception of HbA_1c_. Further, HbA_1c_ was not adjusted for fasting glucose

Age (unit = 8.2 years) was inversely associated with nerve function. Associations with sural nerve SNAP amplitude and tibial nerve CMAP amplitude were most pronounced: *β* = −0.30 (−0.35, −0.25) and *β* = −0.31 (−0.36, −0.25), respectively (Fig. 1b). Further, age was associated with higher VPT (Fig. 2a), but not with neuropathic pain (Fig. 2b).

### Fasting glucose and HbA_1c_

Higher glucose level (unit = 1.6 mmol/l) was associated with worse nerve function for all measures of nerve function and the associations with peroneal and tibial NCV were *β* = −0.17 SD (−0.21, −0.13) and *β* = −0.18 SD (−0.23, −0.14), respectively appeared to be strongest. For HbA_1c_ (unit = 9.6 mmol/mol (3.0%)), similar associations were observed (Fig. 1). Both glucose and HbA_1c_ were also associated with higher VPT and neuropathic pain (Fig. 2).

### Waist circumference

Larger waist circumference (unit = 13.1 cm) was associated with lower sural SNAP (*β* = −0.08 SD [−0.13, −0.02]) and tibial CMAP amplitude (*β* = −0.15 SD [−0.20, −0.10]). Unexpectedly, it was also associated with higher peroneal CMAP amplitude and NCV (Fig. 1b). Further, waist circumference was associated with higher VPT, *β* = 0.08 SD (0.04, 0.13) (Fig. 2a), but not with neuropathic pain (Fig. 2b).

### Triacylglycerol, HDL- and LDL-cholesterol, and lipid-modifying medication

Higher levels of triacylglycerol (unit = 0.9 mmol/l) were not associated with nerve function (Fig. 1a, b). However, triacylglycerol was associated with lower tibial nerve function in model 1 (ESM Fig. 2). HDL-cholesterol (unit = 0.5 mmol/l) was not associated with better nerve function (Fig. 1). LDL-cholesterol (unit = 1.0 mmol/l) appeared to be associated with better nerve function. The use of lipid-modifying medication appeared to be associated with lower nerve function in model 1 (ESM Fig. 2), but these associations were attenuated (some even reversed) in the fully adjusted models. Similarly, lipid-modifying medication was associated with higher VPT and neuropathic pain, but not in fully adjusted models (Fig. 2).

### Systolic blood pressure and antihypertensive medication

Higher systolic blood pressure (unit = 17.8 mmHg) was not associated with worse nerve function. The use of antihypertensive medication was associated with lower nerve function (Fig. 1a), specifically with peroneal CMAP amplitude and NCV: *β* = −0.13 (−0.23, −0.03) and *β* = −0.16 (−0.25, −0.07), respectively (Fig. 1b). Blood pressure and use of antihypertensive medication were not associated with VPT or neuropathic pain in fully adjusted models (Fig. 2).

### Inflammation

Inflammation (unit = *z* score) was associated with worse nerve function: *β* = −0.04 (−0.07, 0.00) (Fig. 1a). Associations of inflammation with higher VPT and neuropathic pain were observed, but were not statistically significant in fully adjusted models (Fig. 2a, b). However, an interaction effect of diabetes status was observed, and therefore, in ESM Fig. 3, associations with individual inflammation markers are presented stratified on the presence of type 2 diabetes. Inflammation was only associated with lower nerve function and VPT in those with type 2 diabetes.

### Smoking

Current smoking (vs never smoking) was associated with lower nerve function: *β* = −0.11 SD (−0.17, −0.04) (Fig. 1a). Former smokers also had lower peroneal NCV: *β =* −0.12 (−0.20, −0.05) (not shown). Smoking was also associated with higher VPT (*β* = 0.17 [0.06, 0.28]) and neuropathic pain (OR 2.13 [1.38, 3.29]) (Fig. 2).

### Absent sural response

The associations between cardiometabolic risk factors and absent sural nerve response (*n* = 165) are shown in ESM Fig. 4. We observed greater odds for an absent sural nerve response for higher age, fasting glucose, Hb1_Ac_ and waist circumference, consistent with the associations observed above.

### Diabetes status

**Fig. 3:**
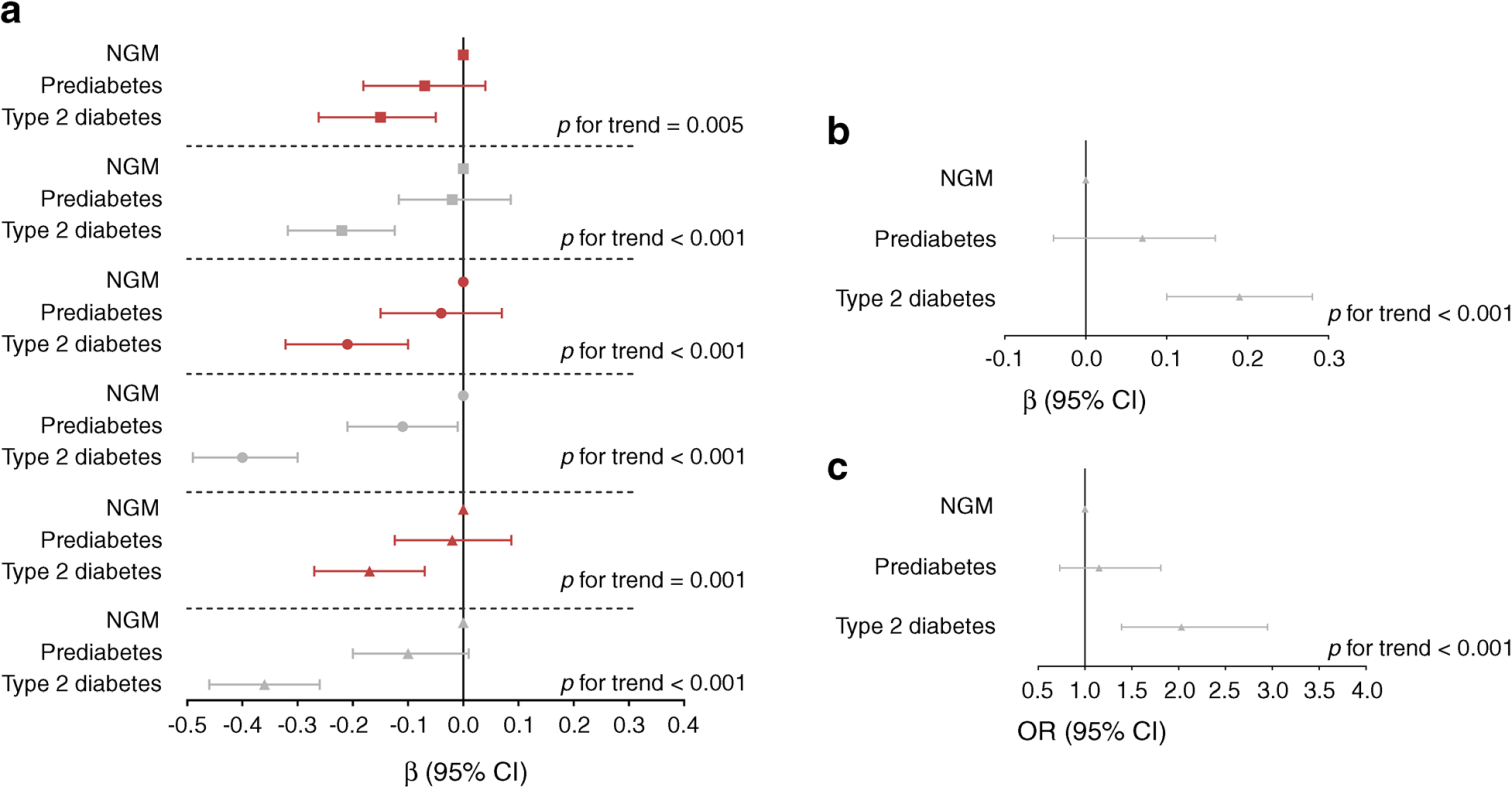
Standardised associations (expressed as *β* [or OR for outcome neuropathic pain] with 95% CIs) of prediabetes and type 2 diabetes with (**a**) nerve function, (**b**) VPT and (**c**) neuropathic pain, adjusted for age, sex, height, skin temperature, education, smoking, alcohol consumption, mobility, CVD (history), kidney function, waist circumference and inflammation. The *p* values indicate linear trend analysis among NGM, prediabetes and type 2 diabetes. Red squares represent sural SNAP amplitude, grey squares represent sural NCV, red circles represent peroneal CMAP amplitude, grey circles represent peroneal NCV, red triangles represent tibial CMAP amplitude and grey triangles represent tibial NCV

Type 2 diabetes was associated with worse nerve function (Fig. 3a). Further, prediabetes appeared to be associated with worse nerve function, although this was only statistically significant for peroneal NCV: *β* = −0.11 SD (−0.21, −0.01). Nonetheless, linear trend analyses showed a consistent trend across NGM, prediabetes and type 2 diabetes; *p* < 0.01 for all measures of nerve function. Type 2 diabetes was also associated with higher VPT (*β* = 0.19 SD [0.10, 0.28]) and neuropathic pain (OR 2.03 [1.39, 2.95]) (Fig. 3b and Fig. 3c, respectively). Further, a trend across NGM, prediabetes and type 2 diabetes was seen for VPT and neuropathic pain (both *p* < 0.001).

### Analyses stratified on diabetes status

In ESM Figs 5 and 6, associations with nerve conduction measures are shown stratified on type 2 diabetes. Overall, results were similar between those with and those without type 2 diabetes. Fasting glucose and HbA_1c_ were not, whereas waist circumference was, associated with VPT (ESM Fig. 7) and neuropathic pain (ESM Fig. 8) in those without type 2 diabetes.

### The metabolic syndrome

Presence of the metabolic syndrome was associated with worse sural SNAP amplitude, tibial CMAP amplitude and tibial NCV: *β* = −0.13 SD (−0.22, −0.04), *β* = −0.15 SD (−0.23, −0.06) and *β* = −0.09 SD (−0.17, 0), respectively (ESM Fig. 9). A linear trend was observed across 0–2, 3, 4 and 5 criteria of the metabolic syndrome for four out of six measures of nerve function. Similarly, a trend was seen with increasing number of criteria for the metabolic syndrome and higher VPT (*p =* 0.05) and neuropathic pain (*p* = 0.027).

In additional analyses, we replaced fasting glucose with 2 h post-load OGTT values (*n* = 2252). These associations appeared somewhat weaker compared with fasting glucose or HbA_1c_ (ESM Fig. 10). Further, we substituted HDL-cholesterol with total-to-HDL-cholesterol ratio. This yielded similar findings. Substituting systolic with diastolic blood pressure or with blood pressure values derived from 24 h measurement also resulted in similar findings (ESM Fig. 10).

## Discussion

To our knowledge, this is the largest population-based study examining mutually independent associations of individual cardiometabolic risk factors with peripheral motor and sensory nerve function using electrophysiological techniques as well as clinical measures such as VPT and neuropathic pain. Older age, higher glucose levels, HbA_1c_, antihypertensive medication, inflammation and smoking were associated with worse sensory and motor nerve function, without any major differences between these two types of nerves. By and large, the same patterns were seen for VPT and neuropathic pain, except that older age and higher waist circumference were more strongly associated with a poorer VPT and there was no association of age and neuropathic pain. These associations were similar for men and women. While type 2 diabetes was, as expected, clearly and consistently associated with worse nerve function and neuropathic pain, trend analyses showed that prediabetes also appeared to be associated with worse nerve function and neuropathic pain.

Previous studies on the relation between prediabetes and nerve function were inconsistent in their findings, which may be due to discrepancies in defining neuropathy or by dichotomising neuropathy as outcome [6–10]. We used continuous, electrophysiological measures of large-fibre nerve function, in different type of nerves (sensory and motor), as primary outcome in order to detect changes at an early stage. In addition, we studied VPTs (a clinical measure of large-fibre dysfunction) and neuropathic pain, which is more related to small-fibre dysfunction. Higher levels of fasting glucose and HbA_1c_, even within the normal range, were associated with lower nerve function. Associations with post-load glucose appeared to be somewhat weaker, which was in line with the Monitoring of Trends and Determinants in Cardiovascular Disease (MONICA)/Cooperative Research in the Region of Augsburg (KORA) study [28]. In contrast, in participants without diabetes, fasting and post-load glucose were not associated with VPT and neuropathic pain.

In accordance with our results, waist circumference (or obesity) has previously been associated with neuropathy and diminished nerve function in people with and without diabetes [14, 20, 29, 30]. This effect might be mediated by low-grade inflammation, as inflammation is associated with diminished nerve function in patients with diabetes [31] and in the general population [32, 33]. Interestingly, in our study, inflammation was associated with diminished nerve conduction and higher VPT only in people with type 2 diabetes, suggesting that inflammation is a consequence of long-term metabolic damages that start before overt diabetes and is not a risk factor that initiates large-fibre damage. However, once present, neuro-inflammation might contribute to further progression of nerve damage. Our results on neuropathic pain are partially in line with the higher circulating Il-6 levels in painful diabetic neuropathy as reported in the KORA F4 study, although we observed no association with sICAM-1 [34]. To further delineate the role of inflammation in the development of small-fibre damage, objective techniques such as corneal confocal microscopy or skin biopsy will be needed.

The metabolic syndrome has also been associated with diminished nerve function [13–15, 17, 20, 30, 35]. However, this is not unexpected as individual components of the metabolic syndrome (glucose, waist circumference and, to a lesser extent, antihypertensive medication) were associated with worse nerve function.

Results of cholesterol and blood pressure (and, to a lesser extent, triacylglycerol) should be interpreted with caution, as over one-third of the population used lipid-lowering and/or antihypertensive medication. In general, hypertension and hypercholesteraemia are treated early in the Netherlands. Consequently, in this relatively healthy and well-treated population, ranges of lipids and blood pressure might be too narrow to observe associations. Nevertheless, antihypertensive medication (which suggests a history of exposure to hypertension) was associated with lower nerve function and, to a lesser extent, with neuropathic pain. Hypertension may affect nerve function by damage of the (nerve) microcirculation. Moreover, as statin use is common in the treatment of diabetes, fasting LDL-cholesterol levels were actually lower in people with type 2 diabetes, compared with people with NGM. This may explain the unexpected finding that LDL-cholesterol was associated with better nerve function. In the Addition study, lower LDL levels were associated with a higher risk of developing diabetic polyneuropathy, and also these authors could not exclude an effect of statins in their analyses [29].

We used electrophysiology, enabling us to detect on a continuous scale differences in large-fibre nerve function that cannot be detected on clinical examination, and studied both motor and sensory nerves. In contrast to earlier studies, we could not observe a difference in the associations of cardiometabolic risk factors with sural or motor nerve function [19, 36]. Partly, this may be due to 165 cases of undetectable sural nerve response in our study. As an undetectable response indicates poor nerve function, this may have led to underestimation in effect size of the associations studied. Further, due to the cross-sectional design of our study, we cannot exclude that cardiovascular risk factors impact on the sural nerve at an earlier stage. For this we need longitudinal data.

Previous studies have indicated that axonal damage (typically reflected as lower CMAP or SNAP amplitudes) is more common in diabetes than demyelinating damage (typically reflected by lower NCV) [36]. Indeed, age and waist circumference had higher magnitudes of associations with low CMAP and SNAP amplitudes as compared with NCV, but these differences were not statistically significant. Thus, whether different cardiometabolic risk factors may affect nerve axons or myelin differentially is unclear from our results, but, if present, such a differential effect seems limited. Moreover, we did not examine small fibres with objective measures and it has been suggested that obesity/hyperlipidaemia and hyperglycaemia may have differential effects on small vs large nerve fibres [37].

A complex interplay between several mechanisms including hyperglycaemia, lipotoxicity, oxidative stress and inflammation is thought to play a central role in the pathogenesis of (diabetic) neuropathy [38]. We recently reported that microvascular function was diminished not only in people with type 2 diabetes, but also with prediabetes [39]; age, smoking and prior exposure to hypertension and dyslipidaemia, and in particular higher levels of glucose (also in the normal range), were all associated with microvascular function [40]. Observations in the current study are in line with these results, suggesting similar risk factors for generalised microvascular damage and early-stage nerve damage. Most likely, preventive or therapeutic measures that target all of these risk factors may be clinically beneficial. However, in contrast to several other microvascular complications, intensive blood glucose control had only a very modest effect in preventing large-fibre neuropathy in type 2 diabetes [41], and also multi-modal interventions, such as in the STENO-2 [42] or the Look AHEAD (Action for Health in Diabetes) studies [43], seemed unsuccessful. Hence, prevention of large-fibre neuropathy should probably start at the earliest signs of diabetes, maybe even in the prediabetic stage, and the role of inflammation in the progression of subclinical to clinical neuropathy should be further explored. Prediabetes is also associated with abnormalities of the central nervous system [44], and it remains to be determined whether the risk factors for central nerve abnormalities are the same as those for peripheral nerves.

Strengths of this study include the use of nerve conduction testing as an objective measure of nerve function that provides insight into nerve damage at very early stages. We investigated different types of nerve and anatomical parts of the nerve and we also included clinical measures in a large, population-based study of adults (aged 40–75 years). Lastly, our statistical models were mutually adjusted and adjusted for many potential confounders. Nonetheless, residual confounding by non-measured factors may still have occurred. Other limitations include its cross-sectional design, and thus inferences regarding causality should be made with caution. As we did not observe major differences between the individual nerves, we summarised our electrophysiological findings in a sum-score, but this should be viewed as a post hoc analysis. The clinical relevance of the observed associations should be investigated in future studies. Further, waist circumference is a crude measure for adiposity and the underlying biological mechanisms explaining the associations between waist circumference and nerve function should be scrutinised. Finally, the inclusion of a relatively healthy population in the Maastricht Study and the exclusion of participants with incomplete assessments of nerve function may have resulted in selection bias, as these participants were older and more often had diabetes. This may have led to an underestimation of the associations observed.

In conclusion, in adults aged 40–75 years, blood glucose (fasting glucose or HbA_1c_), even in the non-diabetic range, was most consistently associated with (sensorimotor) peripheral nerve function and neuropathic pain. Similarly, those with type 2 diabetes, and to a lesser degree those with prediabetes, had worse nerve function. A larger waist circumference, smoking and use of antihypertensive medication (suggestive of history of exposure to hypertension), independent of glucose and other risk factors, were associated with worse nerve function, and similar patterns were observed with VPT and neuropathic pain. The association with low-grade inflammation was most pronounced in participants with type 2 diabetes. These results imply that early-stage nerve damage may result not only from glycaemic damage, but also from other cardiometabolic risk markers. Consequently, multifactorial approaches should be considered in the prevention of neuropathy, rather than a sole focus on blood glucose.

## Electronic supplementary material

ESM (PDF 1.25 mb)

## Data Availability

Data are unsuitable for public deposition due to ethical restrictions and privacy of the participant data according to the study protocol approved by the institutional medical ethical committee (Medisch-ethische toetsingscommissie azM/UM, NL31329.068.10) and the Minister of Health, Welfare and Sports of the Netherlands (Permit 131088-105234-PG). Data are available from the Maastricht Study for any interested researcher who meets the criteria for access to confidential data. The Maastricht Study Management Team (research.dms@mumc.nl) may be contacted to request data.

## Abbreviations

CMAP: Compound muscle action potential
NCV: Nerve conduction velocity
NGM: Normal glucose metabolism
sICAM-1: Soluble intercellular adhesion molecule-1
SNAP: Sensory nerve action potential
VPT: Vibration perception threshold
