# Supplementary material for: Cardiometabolic risk factors as determinants of peripheral nerve function: the Maastricht Study
Source: Diabetologia. 2020 Jun 15;63(8):1648–58. doi: 10.1007/s00125-020-05194-5 (PMC7351845; doi:10.1007/s00125-020-05194-5)
Supplement: Supplementary file 1 — (PDF 1.25 mb) [file 125_2020_5194_MOESM1_ESM.pdf]

ESM; details of methods

Population

We used data from the Maastricht Study, an observational prospective population-based cohort study. The rationale and methodology have been described previously [21]. In brief, the study focuses on the aetiology, pathophysiology, complications, and comorbidities of type 2 diabetes and is characterized by an extensive phenotyping approach. Eligible for participation were all individuals aged between 40 and 75 years and living in the southern part of the Netherlands. Participants were recruited through mass media campaigns and from the municipal registries and the regional Diabetes Patient Registry via mailings. Recruitment was stratified according to known type 2 diabetes status, with an oversampling of individuals with type 2 diabetes, for reasons of efficiency. The present report includes cross-sectional data from 3451 participants, who completed the baseline survey between November 2010 and September 2013. The study complies with the Declaration of Helsinki and has been approved by the institutional medical ethical committee (NL31329.068.10) and the Minister of Health, Welfare and Sports of the Netherlands (Permit 131088-105234-PG). All participants gave written informed consent.

The metabolic syndrome

The metabolic syndrome was defined according to the Adult Treatment Panel (ATP) III guidelines by the presence of 3 or more of the following criteria: (1) waist circumference ≥102 cm for men and ≥88 cm for women; (2) serum triglyceride level ≥1.7 mmol/l; (3) HDL cholesterol level <1.03 mmol/l for men and <1.30 mmol/l for women; (4) fasting glucose level ≥5.6 mmol/l or use of antidiabetic medications (insulin or oral agents); or (5) systolic blood pressure ≥130 mmHg and/or diastolic blood pressure ≥85 mmHg, and/or use of antihypertensive medications.

Cardiovascular disease history

Cardiovascular disease history was defined as a history of any of the following conditions: myocardial infarction, cerebrovascular infarction or haemorrhage, percutaneous artery angioplasty of, or vascular surgery on, the coronary, abdominal, peripheral or carotid arteries.

Statistical analyses

All continuous risk factors and the six outcomes of nerve function were standardised to z scores (with a mean of 0 and an SD of 1) in order to compare the magnitudes of observed associations between all risk factors and outcomes. Consequently, in the regression models, the unit increase for every continuous risk factor is similar to its SD, as presented in table 1 for the normally distributed variables (e.g. 8.2 years for age). For the variables with skewed distribution the unit increase is also the SD, but this is not provided in table 1.

ESM table 1: population characteristics of included and excluded population

	Included with sural response (n=2236)	Included, but absent sural response (n=165)	Excluded, incomplete EMG data (n=462)	Excluded, no EMG performed (n=304)	Excluded, missing covariates (n=284)	
Age (years)	59.0 (8.2)	63.5 (6.7)[a]	61.2 (8.5)[a]	61.7 (7.9)[a]	59.6 (8.3)	
BMI (kg/m^2)	26.6 (4.1)	28.7 (5.4)[a]	28.0 (5.3)[a]	27.7 (5.0)[a]	27.5 (5.0)[a]	
Sex (% men)	50.0	66.1	55.2	49.3	50.7	[b]
Current smoker (%)	12.8	12.1	17.1	13.0	19.8	[b]
History of CVD (%)	15.4	23.6	22.5	15.3	14.3	[b]
Type 2 diabetes (%)	23.7	46.1	36.5	37.1	35.7	[b]
Glucose lowering medication (%)	17.8	35.2	32.5	32.9	35.6	[b]
Antihypertensive medication (%)	36.2	55.8	48.8	43.8	42.0	[b]
Lipid lowering medication (%)	33.4	46.1	43.8	40.8	39.1	[b]

[a] indicates statistically significant (p<0.05) compared with the group included with sural response (n=2236).

[b] indicates statistically significant (p<0.05) between groups.

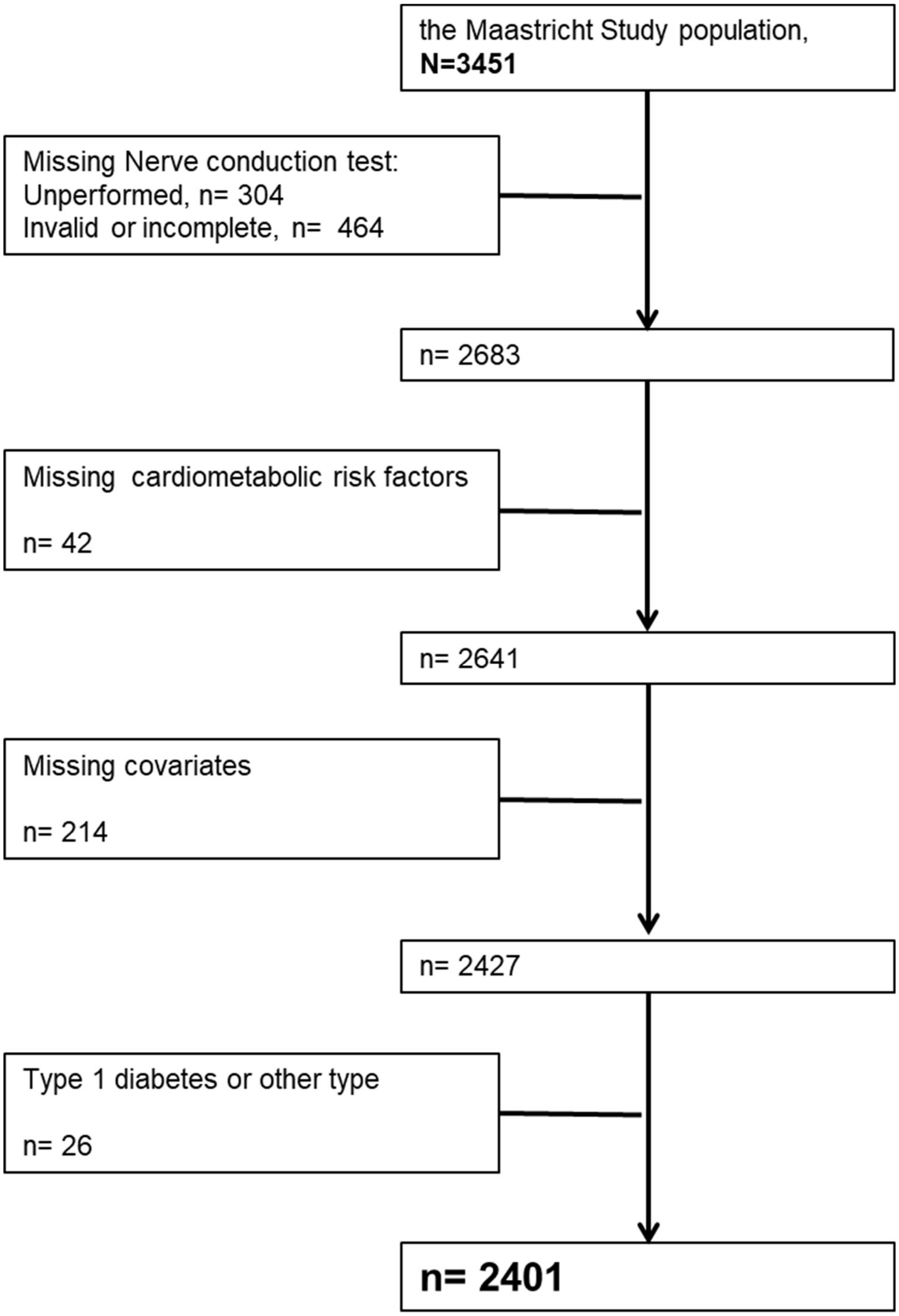

ESM Figure 1: Flow chart of the study population.

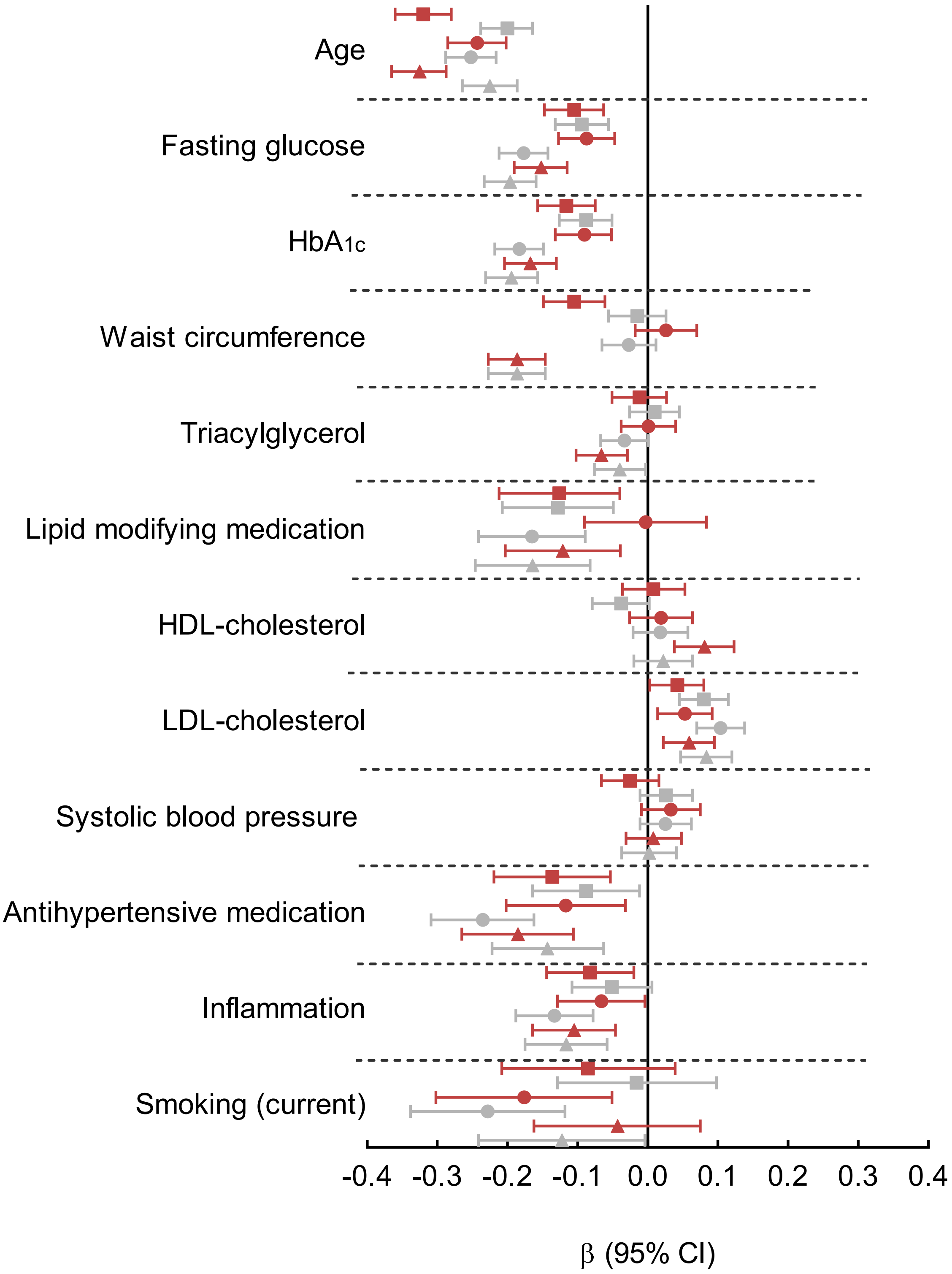

ESM Figure 2: Standardized associations (expressed as *β* with 95% confidence intervals) of cardio-metabolic risk factors and nerve function. Associations were adjusted for sex, height, age (with the exception of associations of age), educational level, and skin temperature (model 1). Red squares represent sural SNAP amplitude, grey squares represent sural NCV, red circles represent peroneal CMAP amplitude, grey circles represent peroneal NCV, red triangles represent tibial CMAP amplitude, grey triangles represent tibial NCV

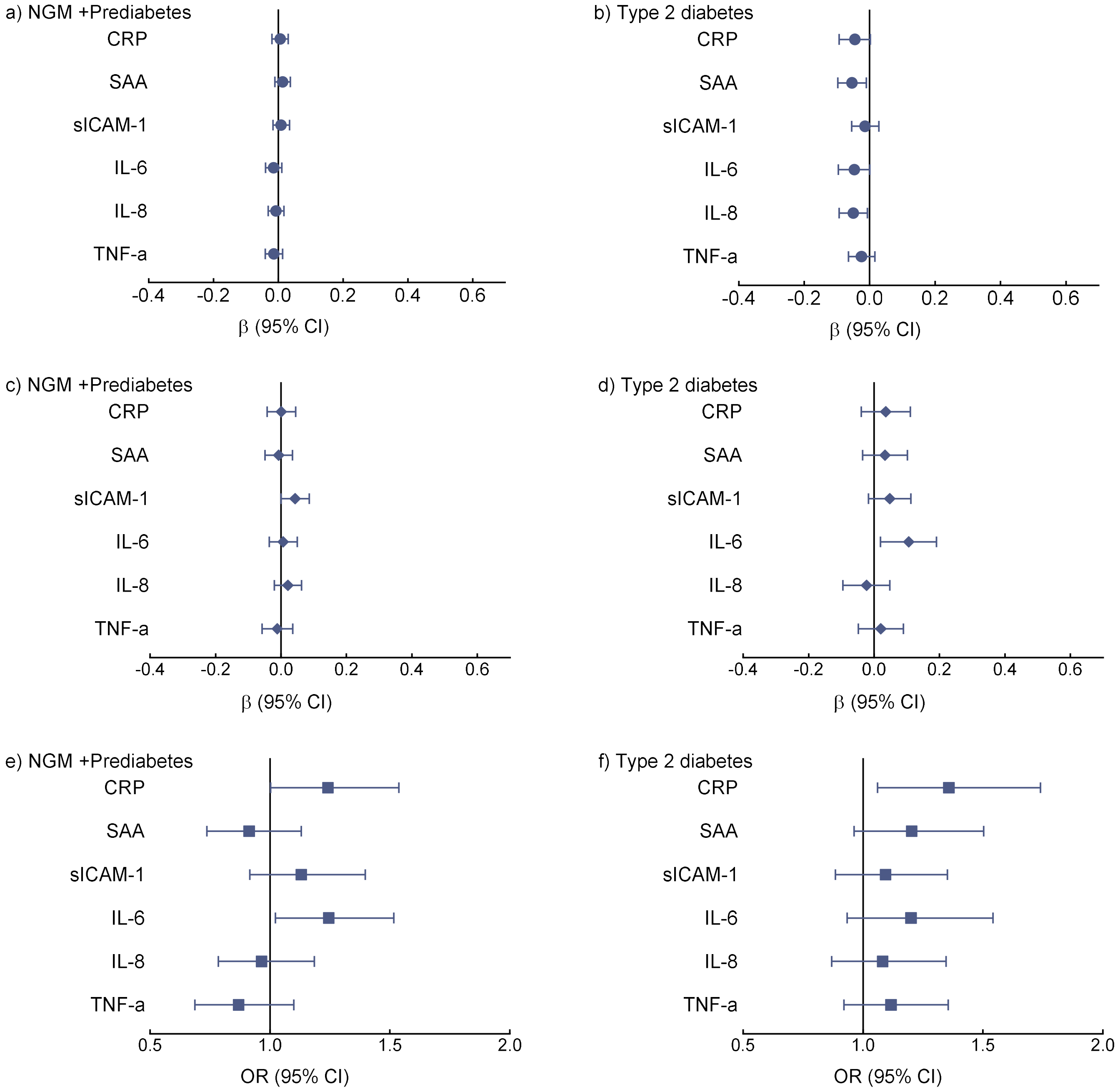

ESM Figure 3: Standardized associations (expressed as *β* with 95% confidence intervals) of individual markers of inflammation and a) and b) sum-score of nerve function, c) and d) vibration perception threshold and e) and f) neuropathic pain stratified by diabetes status; left panels (a, c, e) show associations for people without type diabetes (NGM and prediabetes), right panels (b, d, f), show associations for people with typ2 diabetes. All associations were adjusted for sex, height, educational level, skin temperature, alcohol intake, mobility limitations, CVD (history), and kidney function. In addition, all associations were adjusted for each of the other risk factors with multivariate regression, with the exception of HbA_{1c}. Further, HbA_{1c} was not adjusted for fasting glucose.

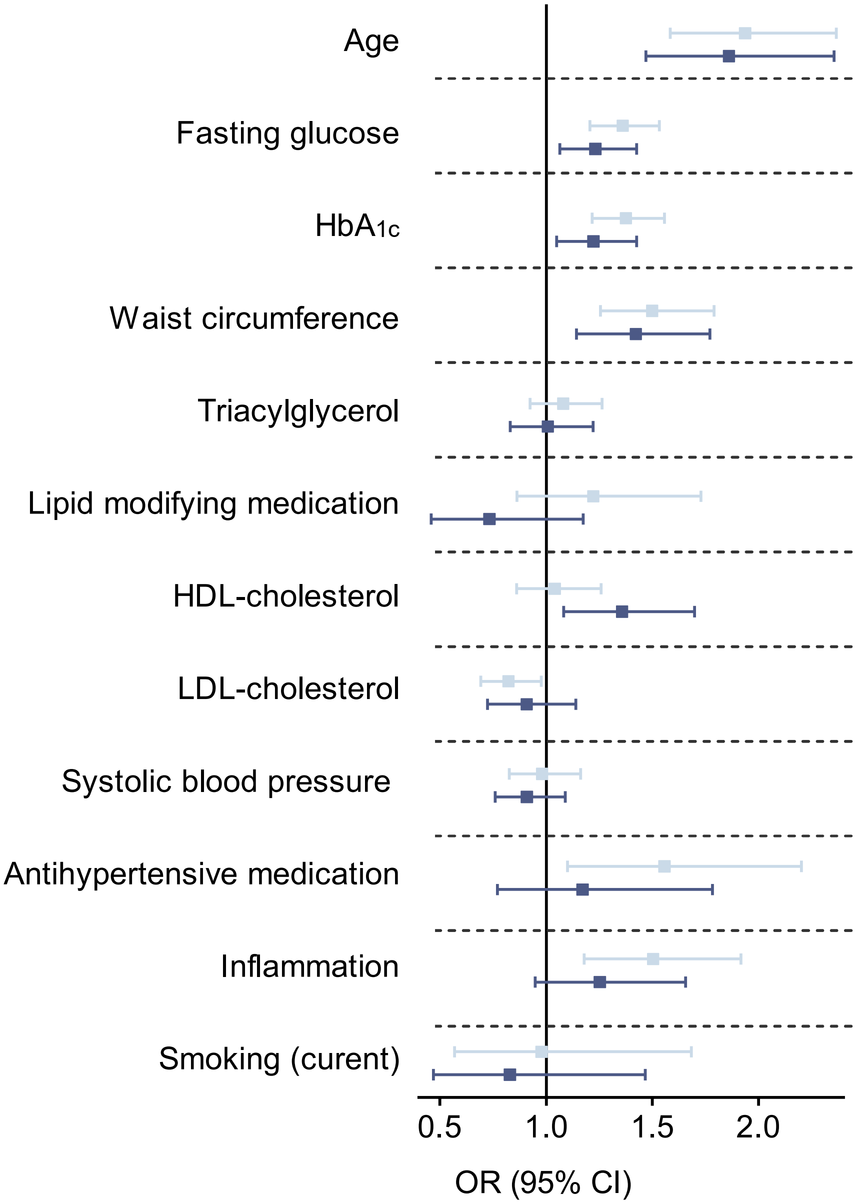

ESM Figure 4: Associations of standardized cardiometabolic risk factors and undetectable sural nerve function, expressed as odds ratios (OR) with 95% confidence intervals. Model 1 (light blue) was adjusted for age, sex, height, educational level, and skin temperature. Associations in model 2 (dark blue) were additionally adjusted for smoking, alcohol consumption, mobility limitations, CVD (history), and kidney function. In addition, all associations in model 2 were adjusted for each of the other risk factors with multivariate regression, with the exception of HbA1c. Further, HbA1c was not adjusted for fasting glucose..

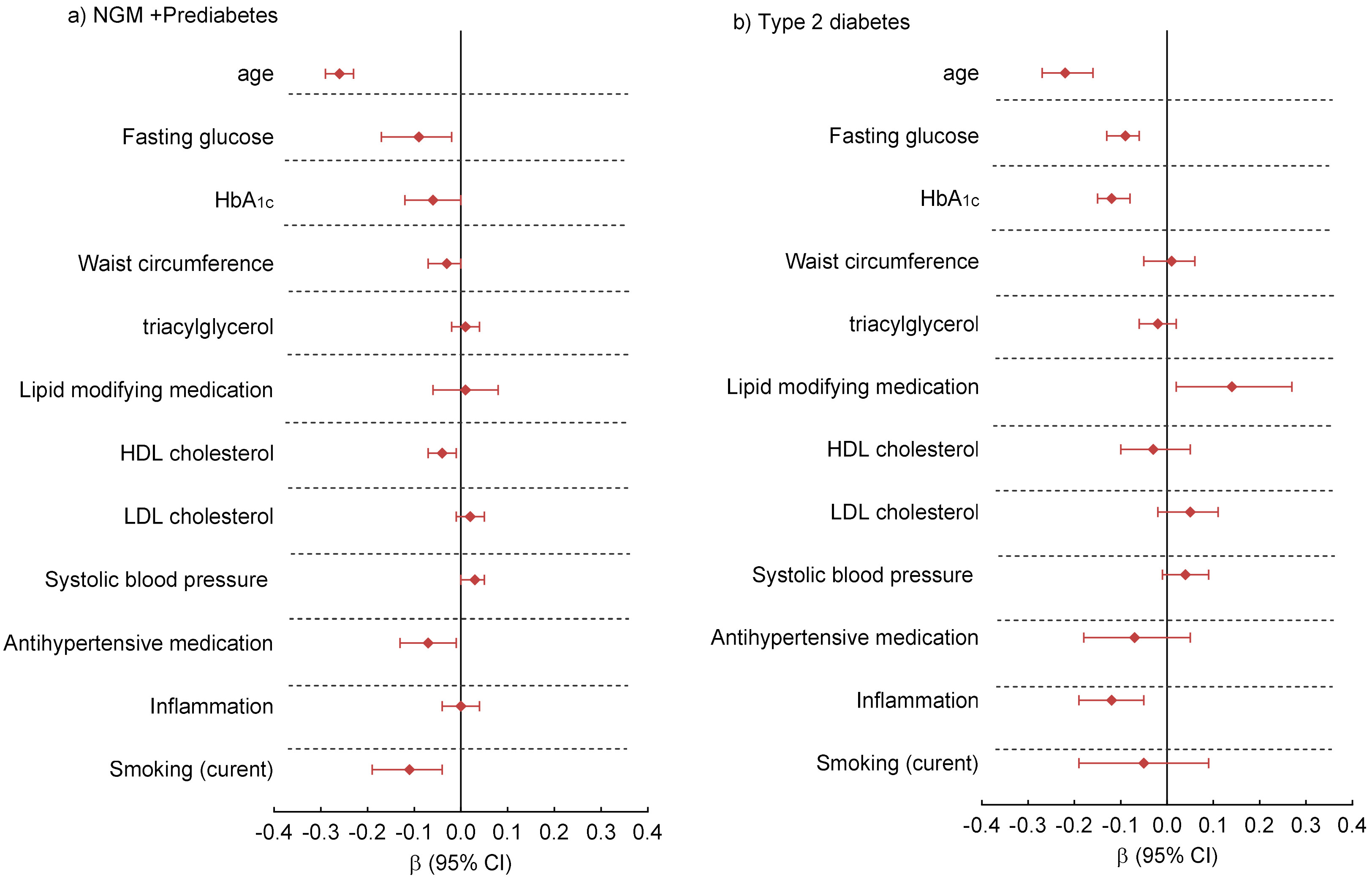

ESM Figure 5: Standardized associations (expressed as *β* with 95% confidence intervals) of cardio-metabolic risk factors and sum-score of nerve function stratified by diabetes status; left panel (a) shows associations for people without type diabetes (NGM and prediabetes), right panel (b), shows associations for people with typ2 diabetes. All associations were adjusted for sex, height, educational level, skin temperature, alcohol intake, mobility limitations, CVD (history), and kidney function. In addition, all associations were adjusted for each of the other risk factors with multivariate regression, with the exception of HbA_{1c}. Further, HbA_{1c} was not adjusted for fasting glucose.

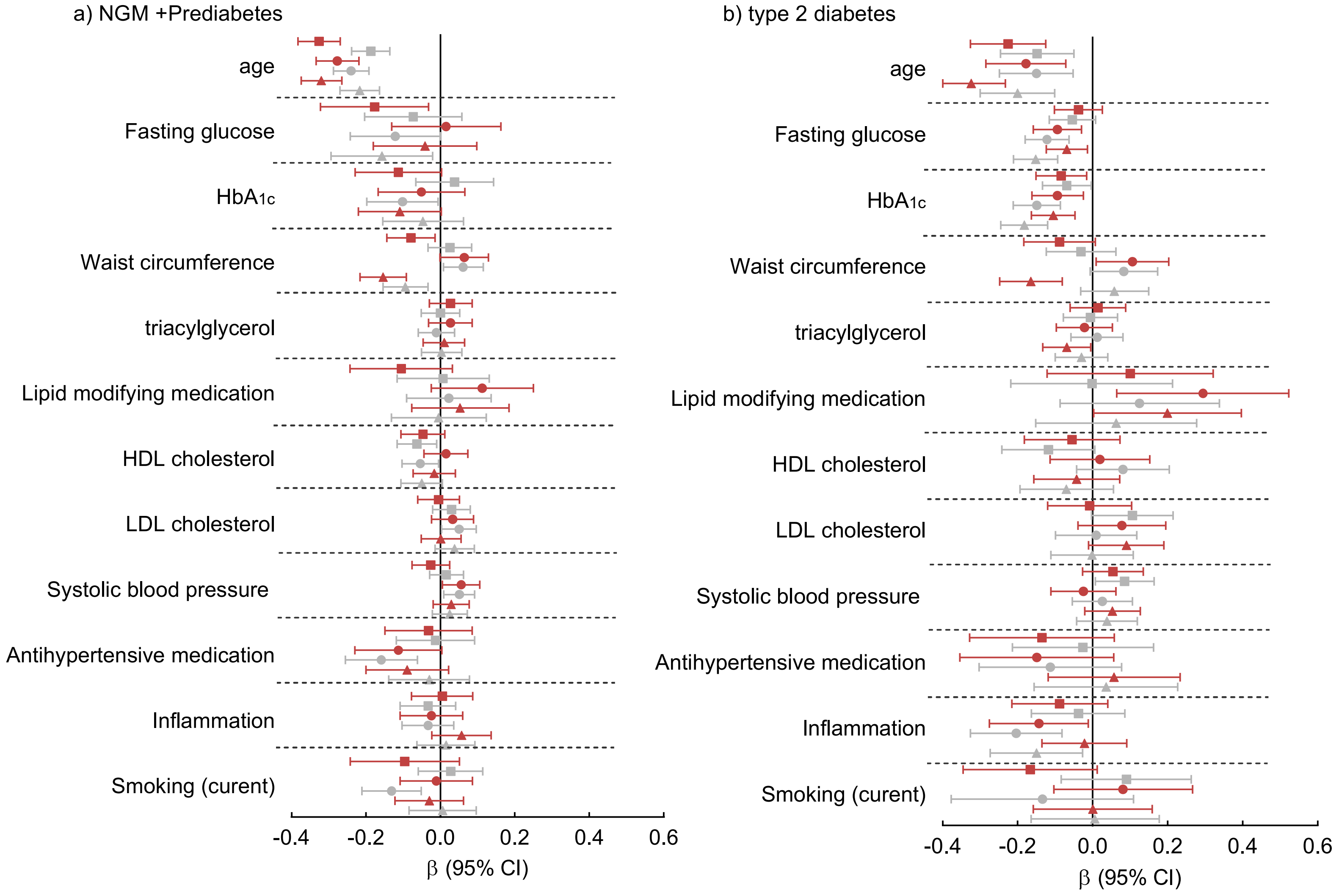

ESM Figure 6: Standardized associations (expressed as β with 95% confidence intervals) of cardio-metabolic risk factors and nerve function stratified by diabetes status; left panel (a) shows associations for people without type diabetes (NGM and prediabetes) , right panel (b), shows associations for people with typ2 diabetes. All associations were adjusted for sex, height, educational level, skin temperature, alcohol intake, mobility limitations, CVD (history), and kidney function. In addition, all associations were adjusted for each of the other risk factors with multivariate regression, with the exception of HbA_{1c}. Further, HbA_{1c} was not adjusted for fasting glucose. Red squares represent sural SNAP amplitude, grey squares represent sural NCV, red circles represent peroneal CMAP amplitude, grey circles represent peroneal NCV, red triangles represent tibial CMAP amplitude, grey triangles represent tibial NCV.

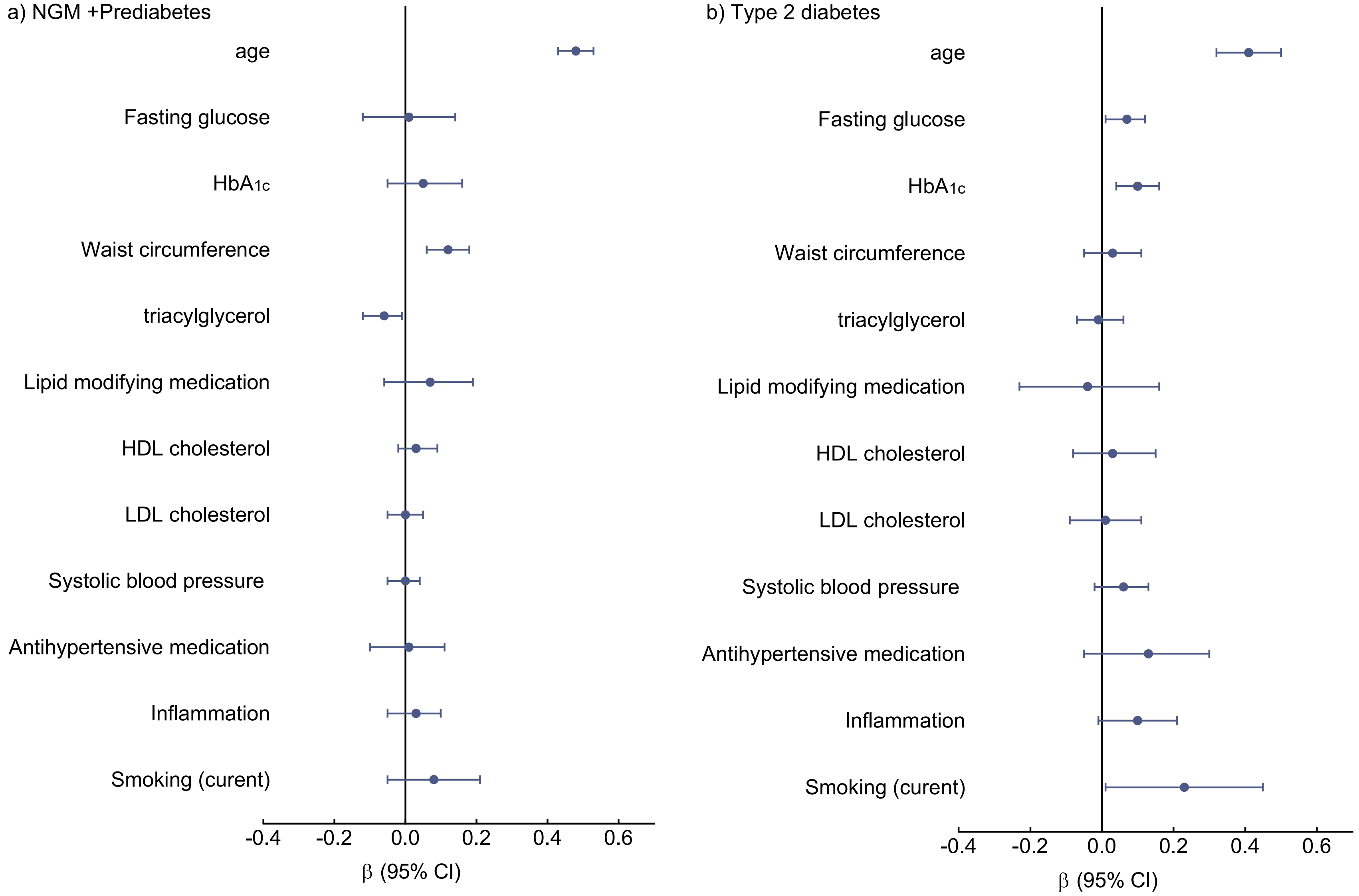

ESM Figure 7: Standardized associations (expressed as *β* with 95% confidence intervals) of cardio-metabolic risk factors and vibration perception threshold stratified by diabetes status; left panel (a) shows associations for people without type diabetes (NGM and prediabetes), right panel (b), shows associations for people with typ2 diabetes. All associations were adjusted for sex, height, educational level, skin temperature, alcohol intake, mobility limitations, CVD (history), and kidney function. In addition, all associations were adjusted for each of the other risk factors with multivariate regression, with the exception of HbA_{1c}. Further, HbA_{1c} was not adjusted for fasting glucose.

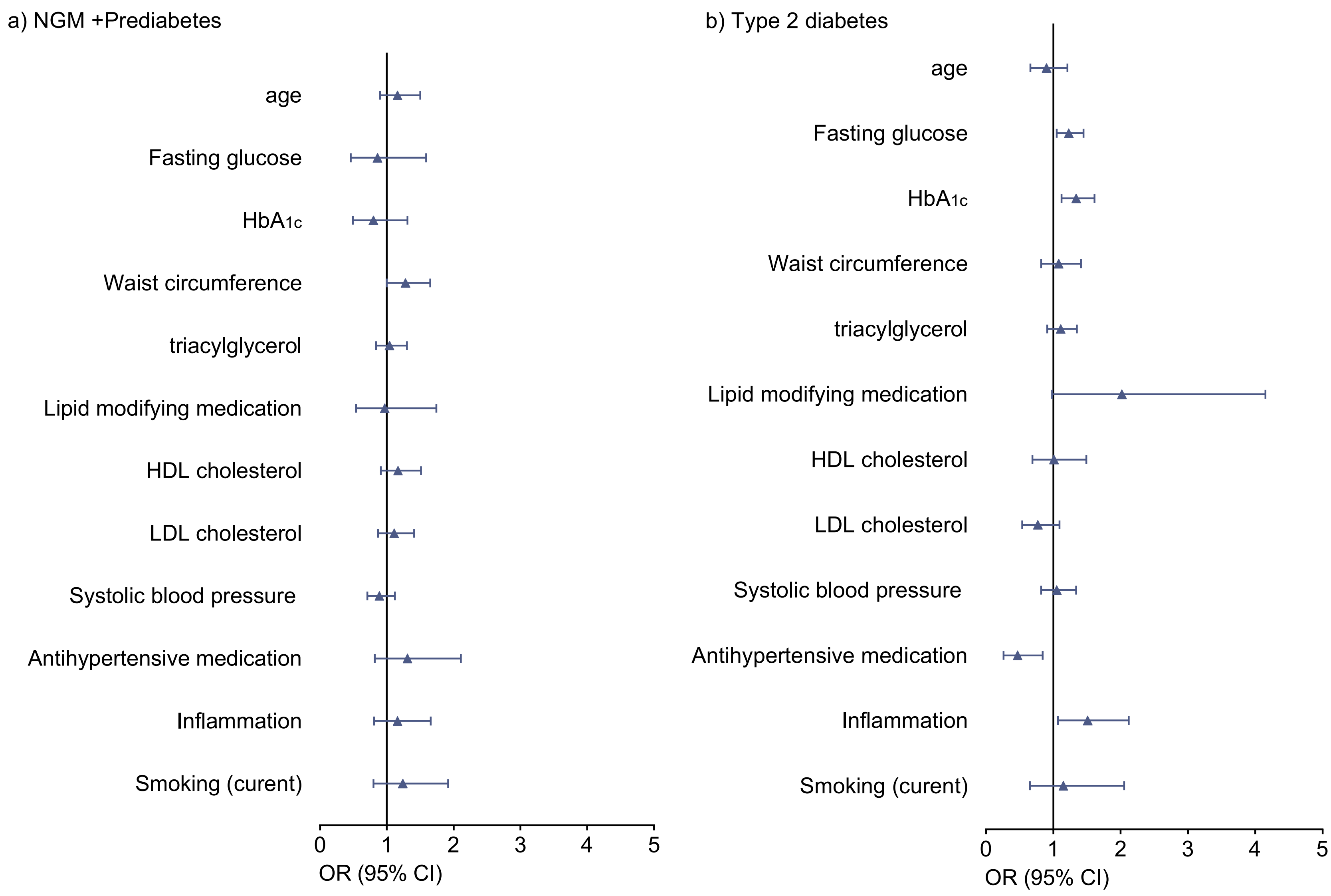

ESM Figure 8: Standardized associations (expressed as odds ratios with 95% confidence intervals) of cardio-metabolic risk factors and neuropathic pain stratified by diabetes status; left panel (a) shows associations for people without type diabetes (NGM and prediabetes), right panel (b), shows associations for people with typ2 diabetes. All associations were adjusted for sex, height, educational level, skin temperature, alcohol intake, mobility limitations, CVD (history), and kidney function. In addition, all associations were adjusted for each of the other risk factors with multivariate regression, with the exception of HbA_{1c}. Further, HbA_{1c} was not adjusted for fasting glucose

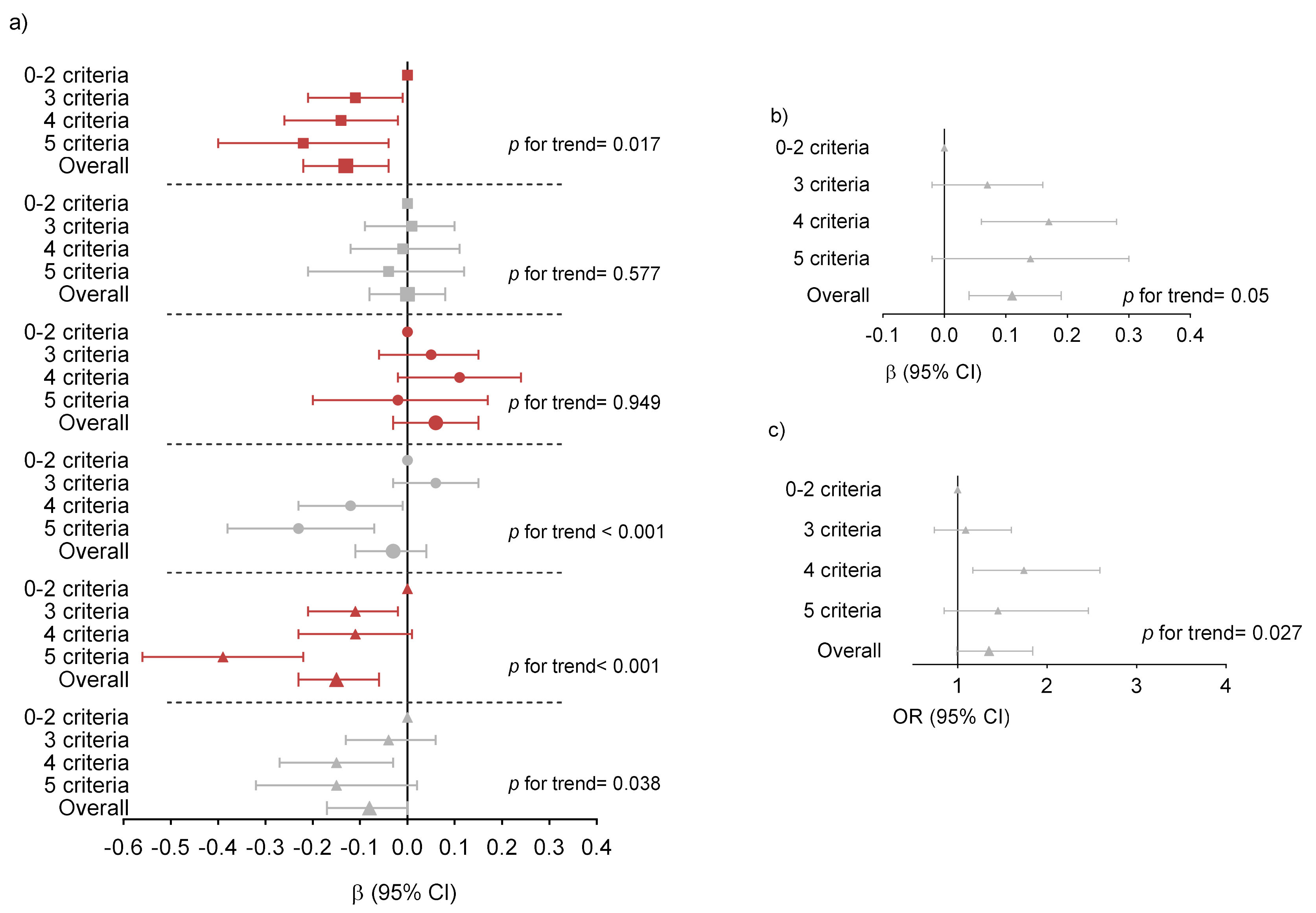

ESM Figure 9: Standardized associations (expressed as *β* or OR with 95%CI) of the metabolic syndrome and number of metabolic syndrome criteria with a) nerve function, b) vibration perception threshold, c) neuropathic pain. Overall indicates the presence of 3 or more metabolic syndrome criteria (larger symbols). All associations were adjusted for age, sex, height, skin temperature, education, smoking, alcohol, mobility, CVD (history), kidney function, and inflammation. *P*-values indicate linear trend analyses among 0-2, 3, 4, 5 criteria of the metabolic syndrome. In a), red squares represent sural SNAP amplitude, grey squares represent sural NCV, red circles represent peroneal CMAP amplitude, grey circles represent peroneal NCV, red triangles represent tibial CMAP amplitude, grey triangles represent tibial NCV

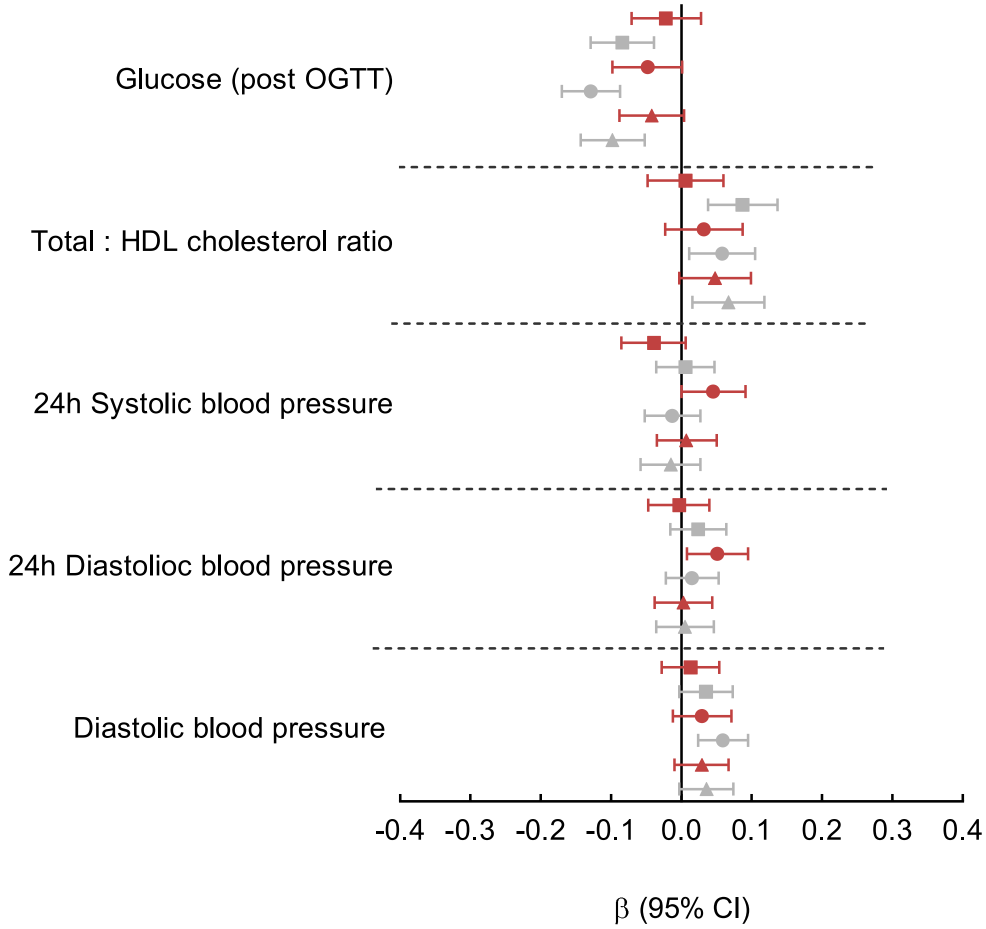

ESM Figure 10: Standardized associations (expressed as β with 95%CI) of (office) diastolic blood pressure and post-load glucose with nerve function. Associations were adjusted for sex, height, age, skin temperature, education, smoking, alcohol, mobility limitations, CVD (history), and kidney function. In addition, all associations were adjusted for each of the other cardiometabolic risk factors shown in Figure 1, (but blood pressure measures not for systolic blood pressure and post-load glucose not for fasting glucose). Red squares represent sural SNAP amplitude, grey squares represent sural NCV, red circles represent peroneal CMAP amplitude, grey circles represent peroneal NCV, red triangles represent tibial CMAP amplitude, grey triangles represent tibial NCV.
